# Why MSM in Rural South African Communities Should be an HIV Prevention Research Priority

**DOI:** 10.1007/s10461-012-0356-1

**Published:** 2012-11-30

**Authors:** John Imrie, Graeme Hoddinott, Sebastian Fuller, Stephen Oliver, Marie-Louise Newell

**Affiliations:** 1 Africa Centre for Health and Population Studies, University of KwaZulu-Natal, PO Box 198, Mtubatuba, KwaZulu-Natal 3935 South Africa; 2 Faculty of Population Health Sciences, Centre for Sexual Health and HIV Research, University College London, London, UK; 3 University College London Institute of Child Health, London, UK

**Keywords:** Rural, MSM, HIV prevention, Research, South Africa

## Abstract

Research into HIV and men who have sex with men’s (MSM) health in South Africa has been largely confined to the metropolitan centres. Only two studies were located making reference to MSM in rural contexts or same-sex behaviors among men in the same. There is growing recognition in South Africa that MSM are not only disproportionately affected by HIV and have been underserved by the country’s national response, but that they contribute significantly to sustaining the high number of new infections recorded each year. We argue that to meet the objectives of the country’s national strategic plan for HIV, STI and TB it is important we know how these behaviours may be contributing to the sustained rural HIV epidemic in the youngest age groups and determine what constitutes appropriate and feasible programmatic response that can be implemented in the country’s public sector health services.

## Introduction

Until recently men who have sex with men (MSM) in Africa were largely overlooked in descriptions, discussions and responses to the continent’s HIV epidemic [1]. However, MSM have recently gained prominence in policy positions of key organizations like UNAIDS and WHO, been recognized as a key group in the national HIV plans of a small number of countries including South Africa, and become a target group for programs funded by major donors [2–7]. This is certainly not before time. In South Africa heterosexual transmission is still the dominant mode of HIV transmission, but evidence suggests the country’s epidemic is more diverse, and that MSM behavior plays an important part [7, 8].

Research from South Africa demonstrates MSM are not only disproportionately affected by HIV, but that MSM behavior contributes significantly to sustaining the high number of new infections recorded each year [8–10]. No accurate estimates of South Africa’s MSM population exist and only one national population survey has attempted to quantify their number. The Human Sciences Research Council’s (HSRC) 2008 National HIV Prevalence, Incidence, Behaviour and Communication Survey found 3.2 % of men self-reported same-sex behavior, giving a population roughly estimated at 750,000 adult males, and of these, an estimated 10 % were HIV-infected [8]. Estimates of HIV prevalence in community samples of MSM from the main cities are generally higher, varying from 10.4 to 47.2 % according to location and sampling method [11–14], while a national modeling exercise estimated that MSM same-sex behaviors contributed approximately 9.2 %, or approximately 34,000 to the total new adult HIV infections in 2010 [7, 9, 10]. In an era of renewed prevention optimism, and a growing arsenal of prevention options, including MSM as a specific priority group is appropriate and timely. However, as previous research has shown, a presumption that MSM can be treated as an undifferentiated and homogenous category could be problematic [15, 16].

## Men Who Have Sex With Men in Rural South African Communities Are Under-Researched and All But Invisible

Research into men who have sex with men and other male–male same-sex behavior in South Africa has been almost totally restricted to community studies in the country’s metropolitan centers—Cape Town, Johannesburg, Pretoria and Durban—with scant consideration of these men and the behavior beyond the urban nexus [11–14, 17]. A database search of the peer-reviewed scientific literature on MSM since 2005 highlights the near total absence of work conducted in South Africa’s towns, rural communities or ‘closed communities’ such as prisons, correctional facilities, boarding schools and colleges [18, 19]. Of nearly 100 papers and abstracts identified, searching PubMed, PsychINFO and Google Scholar using standard MeSH (medical subject headings) terms and key word combinations (men, homosexuality, MSM, gay men, HIV, rural and South Africa), less than a handful made reference to MSM or male–male sexual behavior in rural contexts or ‘closed communities’. Only two studies in young rural men reported male–male sexual behavior and discussed the implications for HIV prevention—one from the Eastern Cape and the other from KwaZulu-Natal [20, 21].

These two studies found that among men that reported same-sex behavior, almost none identified as ‘gay’, or exclusively MSM, and most reported having sexual contact with both men and women [20, 21]. Reporting same-sex behavior was associated with significantly higher levels of gender violence perpetration and coerced sex, including male–female and male–male rape [20]. In the first study in the Eastern Cape, reporting same-sex behavior was associated with significantly greater risk of being HIV-infected [20], while in the KwaZulu-Natal study, it was linked to significantly higher scores on a measure of gender role confusion [21]. This is perhaps not surprising given the concept of MSM is itself debated, and ideas of MSM sexual practices are not necessarily contextually specified or culturally articulated the same way in every situation [16, 20]. As the authors of the first study concluded, what we know about same-sex behavior among young rural men is very limited and “the question of what drives the sexual [HIV] epidemic in the youngest age groups is important” and therefore “understanding this [same-sex sexual behavior] is necessary for developing appropriate programmatic responses … for both young men and their female partners (p. 1,459)” [20].

What both these studies suggest is that men in rural contexts that report male sexual partners, also have female partners and therefore may be more likely to have more lifetime partners overall [22]. The role sex with female partners may have on MSM HIV risks has been studied in other contexts and shown that MSM who also have sex with women have somewhat lower rates of HIV infection [23, 24], research from our setting confirms, that in hyper-endemic rural areas, higher numbers of lifetime partnerships carries significant additional risk for HIV acquisition [25]. The current literature on MSM and same-sex sexual behavior among rural men is inadequate to know the importance of these behaviors, what the effect is of having both male and female partners, or whether men reporting same-sex sexual behavior are important in sustaining the rural HIV epidemic. The answer to these questions is likely to be complex and multi-dimensional [23, 24, 26]. We will not be able to answer any of these questions until we begin to look more closely.

## Reporting Same-Sex Practices Among Young Men in Rural Northern KwaZulu-Natal

However starting to answer this question is not an insurmountable challenge. Our experience in the rural Hlabisa sub-district of northern KwaZulu-Natal demonstrates that while men with same-sex sexual behavior experience maybe deeply hidden in their communities, it may not be difficult to find them in the context of research studies specifically relating to men’s health. The Impilo Yamadoda—Men’s Health Study focused on evaluating strategies to improve recruitment and retention of male participants in HIV prevention trials [27]. In the quasi-experimental phase of this study, 223 young (aged 18–35 years) Zulu men were recruited, randomized, and followed-up over 12 months. They were surveyed on four occasions (baseline, 3, 6 and 12 months follow-up), using questionnaires administered by young trained male fieldworkers from the same communities. At each survey round men were asked about sexual partnerships and behaviors during the previous 3 months. Of the 223 study participants, 7 (3.1 %) reported sex with a male partner (in the previous 3 months) at least once in the four survey rounds. A closer look at the men’s responses highlights why detailed research into the sexual attitudes, lifestyles, sexual practices and experiences of young men in rural communities, men that identify as ‘gay’ and/or MSM, and men that do not, but report same-sex sexual behavior, is needed to ensure an adequate sexual health, HIV prevention and treatment and care response.

The study was conducted in a rural area that includes a large demographic surveillance site where the dynamics and impact of the HIV epidemic on the local area has been carefully studied for more than 10 years (www.africacentre.com). This is important because apart from their reported same-sex sexual behavior, these men were remarkably similar to their counterparts in the study and other young men in the local population [28, 29]. Table 1 compares baseline demographic, HIV-testing and sexual HIV risk characteristics of men according to whether they reported sex with a male partner in the last 3 months at any survey round.

**Table 1:** Baseline demographic, HIV-testing and sexual HIV risk characteristics of men in the Impilo Yamadoda—Men’s Health Study according to reporting of sex with a male partner in the last 3 months at any survey round

	Men who report sex with male partner in last 3 months at any survey round (*n* = 7)	Men who did not report sex with male partner in last 3 months at any survey round (*n* = 216)
Age (Median)	19–25 years (20)	18–35 years (22)
Employment		
Employed	1 (14 %)	7 (16 %)
Full-time education	4 (57 %)	84 (39 %)
Unemployed/other	2 (29 %)	125 (58 %)
HIV tested in last 3 months	1 (14 %)	38 (18 %)
Sexual partnerships		
>1 sexual partner in last 3 months	3 (43 %)	99 (42 %)
≥1 one-time-only sexual partner (during follow-up)	7 (100 %)	165 (76 %)
Condom-use		
Ever condom used	6 (86 %)	206 (95 %)
Condom used with most recent partner	4 (57 %)	156 (72 %)
Condom used with most recent one-time-only partner	4 (57 %)	164 (75 %)
Sexual coercion		
Ever sex unwillingly (forced sex)	1 (14 %)	17 (7 %)
Exchange sex		
Received gifts or money for sex	2 (29 %)	11 (5 %)
Given gifts or money for sex	2 (29 %)	10 (5 %)
HIV risk perception		
Agreed with statement—“My everyday behaviour puts me at risk of acquiring HIV”	1 (14 %)	31 (14 %)

The seven men that reported having had sex with a male partner were similar to young men in the area in relation to other sexual behaviors and HIV risks. None of the seven men reported a male sexual partner in more than one survey round and all reported at least one female partner at some point. Anal and oral sex are not common practices in this population [16, 30–32], and none of the seven reported engaging in anal or oral sex with their male or female partners. The reported condom-use rates of men in the study were also similar to those observed in other research involving young people in this area [33].

It is our belief that the 3.1 % (7/223) of men that reported a recent same-sex sexual partner is an under-report. Our study participants were men living in very traditional rural communities, where same-sex behaviors are not talked about and are often poorly understood [16, 24, 31, 32]. Despite this, these men were able to report same-sex behavior in face-to-face interviews with young trained male fieldworkers from their own community, but possibly because of the stigma attached or lack of understanding, they were not able to describe the behavioral composition of those episodes [13, 15, 16, 26]. The reported 3.1 % MSM prevalence is slightly below the 3.6 % (46/1277) rate of ‘ever sex with a male partner’ reported in the Eastern Cape study and substantially lower than the 15 % ‘sex with a male partner’ found among men who reported that they were sexually active in the last 2 months in the KwaZulu-Natal study [20, 21]. What is significant however is that these findings confirm same-sex sexual behavior exists among these young rural men, whatever form it may take and that these young men also have sex with female partners. What these and the other findings cannot do however is give adequate insight into the extent to which this population practices exclusive MSM behavior, bisexuality or bisexual concurrency, all of which are important to answer our overarching question [7, 12, 13, 26].

## The Case for Making MSM in Rural South African Communities an HIV Research Priority

In light of previous research and our own findings, there is undoubtedly a case for making MSM and other men who practice same-sex sex in towns and rural communities in South Africa an HIV and sexual health research priority. In our view the case rests on three pillars. The first is that the very limited data would seem to suggest men in these communities reporting same-sex behavior are somehow different to the studied MSM populations in South Africa’s urban and peri-urban communities [11–15, 17, 23, 32, 34, 35]. For example, in urban studies more participants report exclusive MSM sexual behavior, as well as a large proportion that report bisexuality or heterosexual-identity and same-sex sexual behaviors [11, 12]. It is unclear from the available research in towns and rural areas whether bisexual practice, and potentially bisexual concurrency, or possibly just rare episodes of same-sex behavior, is the norm or whether it is a matter of choice, necessity or simple opportunity [14, 24–26]. None of the men in our study reported being exclusively MSM, and in other rural samples the numbers are very small; only one participant in Jewkes et al.’s study [20] in the Eastern Cape actually identified as gay. It seems that young rural men that report same-sex experiences do not possess a sense of being ‘gay’, or of MSM identity, but this may be a matter of language [15, 16, 18]. In many cultures intimate sexual activity between men is not spoken about, or possibly only referred to obliquely, with ‘sex’ being a term reserved to describe activity that potentially leads to procreation [16]. This may result in a poor appreciation of the elevated HIV risk associated with some MSM behaviors, or equally, researchers overlooking practices such as mutual masturbation and thigh-sex (*ukusoma*) that do not elevate HIV risk [35, 36]. The form and content of ‘sex’ that occurs between men in these contexts needs to be thoughtfully investigated, because it could also mean MSM engagement strategies premised on at least a degree of MSM identification or acknowledgement, are ineffective with these men [17, 26, 35, 37]. It appears that these young men that report same-sex behavior are like other men in their own communities, subscribing to traditional cultural practices, including for example, traditional circumcision and religious practices [15, 17, 20]. Research among MSM in South Africa’s urban and peri-urban contexts, alongside findings from studies in other settings, indicate that being linked into traditional culture makes self-acceptance, disclosure or discussion of same-sex behavior or attraction, with family, friends and health care providers very difficult [16, 35, 38–40]. Engaging these men with targeted health promotion is almost certainly going to be challenging. Without additional formative research, the extent of these challenges in rural South African communities will remain unquantified.

The second pillar of the case for prioritizing rural MSM involves ensuring that these men profit from the current supportive environment around MSM health. The convergence of international and national HIV policy agendas into ‘a perfect storm’ of resolve, willingness and commitment is a unique opportunity to advance MSM health in South Africa and beyond in the African continent [23, 35]. Although MSM were included in the objectives of South Africa’s previous National Strategic Plan for HIV and STI (2007–2011) [41], according to the end of term report, and by the current Minister of Health, Dr. Aaron Motsoaledi’s own admission, the South African Government’s response “… certainly (hasn’t) done enough to protect this group” and a “renewed focus on HIV treatment and prevention for MSM is critical” [7, 42]. The South African Government’s new National Strategic Plan for HIV, STIs and TB (2012–2016) explicitly recognizes, and indeed places MSM among the key target groups for prevention education and communication interventions [6], while the South African National AIDS Council’s (SANAC) implementation plans commit all its members to realizing the NSP’s priorities within the context of protecting human rights and improving access to justice [43]. These commitments are a window of opportunity to ensure all MSM are part of the agenda, however without more robust research data it is hard to imagine how MSM in rural communities’ needs will be included.

The third pillar of the case is that more research with MSM, and specifically rural MSM is needed to ensure that public health services are able to respond adequately and appropriately. It is well documented that men in general, and MSM specifically, encounter barriers, stigmatizing comments and abuse when accessing and engaging with health services [35, 44–49]. In the context of HIV in South Africa, the situation is often made worse by representations of men as deviant and pervasive cultures of blame such that delayed accessing of health services is common [35, 45–49]. It is hardly surprising, that research looking at MSM’s experiences of using South Africa’s public sector health services finds MSM generally report negative encounters and commonly experience stigma, discrimination, and negative attitudes from health-care workers [46, 49]. To date, in rural communities, MSM have been entirely ignored by public sector health and social services. Initiatives proposed to address this center around training and capacity-building in communicating skills and instilling attitudes needed to engage MSM empathetically [50]. Yet, the available evidence would suggest, MSM in rural communities are likely to present as heterosexually-identified, and even with additional training, clinics are unlikely to provide sufficient confidential opportunity for men to explore their individual needs, or disclose their bisexuality or same-sex attractedness with a sympathetic health care provider. Programming appropriate services and professional capacity-building to provide services for heterosexually-presenting men with same-sex sexual experiences, regular male or occasional male partners will be a challenge for which we have no evidence-base [23, 24]. In support of the objectives of the new NSP, research that explores and better describes the lived experiences of rural MSM is needed to know what level of programming and capacity-building is needed and what level of specialist service provision is feasible in rural primary health care clinics.

However, the limitations of the current research base need not be an insurmountable obstacle to implementing targeted prevention and treatment strategies for MSM including those in rural communities. While we strengthen local research to inform a set of culturally and contextually appropriate programmatic interventions, we can draw on the experience of others and mount an immediate stop-gap response. The US Centers for Disease Control and Prevention’s compendium of interventions for which there is good evidence of effectiveness includes examples specifically targeting rural MSM (www.effectiveinterventions.org). Some of these may be useful, although replication without sufficient consideration of local contextual factors may deliver no improvement in prevention outcomes. There is however a growing literature on community and structural interventions targeting MSM coming from the global South [51, 52], that may possibly be supplemented with experience, expertise and evidence from parts of the global North where large MSM populations live in rural communities [53–55]. Drawing on all of these resources judiciously could provide the stop-gap measures needed immediately.

## Conclusions

Men who have sex with men populations are not homogeneous within or across regions of countries [16, 23, 35]. Although there is an increasingly useful body of work concerning MSM in South Africa, MSM in rural communities have been overlooked and their HIV and other health needs ignored. Yet their existence and some specific HIV prevention issues for this sub-population have been confirmed. An opportunity exists to redress this imbalance with the growing support for human rights based approaches to HIV prevention internationally and among South Africa’s key HIV stakeholders [41, 42, 56]. However, the research base in South Africa needs to expand to inform, develop and better deliver culturally appropriate interventions for these populations. But in the meantime there is an evidence-base of interventions from both resource-rich and resource-limited settings that may be carefully tapped into in order to begin adapting and delivering stop-gap responses. What proves feasible and acceptable in South Africa often provides a basis for replication elsewhere in the continent. Undertaking research among MSM in many parts of Africa is fraught with challenges, legislative obstacles and cultural barriers [23, 35, 57]. In South Africa the hurdles are fewer, and formative research work, evaluation and implementation of prevention and care programs for rural MSM should be relatively easier, without any loss of the demonstration benefit. Challenging as it will be, making MSM in rural South African communities a prevention research priority is a public health, human rights and HIV challenge which we can no longer ignore.
